# Ebola management centre proximity associated with reduced delays of healthcare of Ebola Virus Disease (EVD) patients, Tonkolili, Sierra Leone, 2014–15

**DOI:** 10.1371/journal.pone.0176692

**Published:** 2017-05-01

**Authors:** Georgios Theocharopoulos, Kostas Danis, Jane Greig, Alexandra Hoffmann, Henriette De Valk, Augustine Jimissa, Sumaila Tejan, Mohammed Sankoh, Karline Kleijer, Will Turner, Jay Achar, Jennifer Duncombe, Kamalini Lokuge, Ivan Gayton, Rob Broeder, Ronald Kremer, Grazia Caleo

**Affiliations:** 1 European Programme for Intervention Epidemiology Training (EPIET), European Centre for Disease Prevention and Control (ECDC), Stockholm, Sweden; 2 Institut de Veille Sanitaire, Saint-Maurice, France; 3 Médecins Sans Frontières, Magburaka, Sierra Leone; 4 Manson Unit, Médecins Sans Frontières, London, United Kingdom; 5 State Office for Health and Social Affairs, Berlin, Germany; 6 Postgraduate Training for Applied Epidemiology (PAE), Robert Koch Institute, Berlin, Germany; 7 District Health Management Team, Ministry of Health and Sanitation, Magburaka, Sierra Leone; 8 Operational Centre Amsterdam, Médecins Sans Frontières, Amsterdam, The Netherlands; 9 National Centre for Epidemiology and Population Health, Research School of Population Health, Australian National University, Canbera, Australia; Hokkaido University Graduate School of Medicine, JAPAN

## Abstract

Between August-December 2014, Ebola Virus Disease (EVD) patients from Tonkolili District were referred for care to two Médecins Sans Frontières (MSF) Ebola Management Centres (EMCs) outside the district (distant EMCs). In December 2014, MSF opened an EMC in Tonkolili District (district EMC). We examined the effect of opening a district-based EMC on time to admission and number of suspect cases dead on arrival (DOA), and identified factors associated with fatality in EVD patients, residents in Tonkolili District. Residents of Tonkolili district who presented between 12 September 2014 and 23 February 2015 to the district EMC and the two distant EMCs were identified from EMC line-lists. EVD cases were confirmed by a positive Ebola PCR test. We calculated time to admission since the onset of symptoms, case-fatality and adjusted Risk Ratios (aRR) using Binomial regression. Of 249 confirmed Ebola cases, 206 (83%) were admitted to the distant EMCs and 43 (17%) to the district EMC. Of them 110 (45%) have died. Confirmed cases dead on arrival (n = 10) were observed only in the distant EMCs. The median time from symptom onset to admission was 6 days (IQR 4,8) in distant EMCs and 3 days (IQR 2,7) in the district EMC (p<0.001). Cases were 2.0 (95%CI 1.4–2.9) times more likely to have delayed admission (>3 days after symptom onset) in the distant compared with the district EMC, but were less likely (aRR = 0.8; 95%CI 0.6–1.0) to have a high viral load (cycle threshold ≤22). A fatal outcome was associated with a high viral load (aRR 2.6; 95%CI 1.8–3.6) and vomiting at first presentation (aRR 1.4; 95%CI 1.0–2.0). The opening of a district EMC was associated with earlier admission of cases to appropriate care facilities, an essential component of reducing EVD transmission. High viral load and vomiting at admission predicted fatality. Healthcare providers should consider the location of EMCs to ensure equitable access during Ebola outbreaks.

## Introduction

On August 8, 2014 the World Health Organization (WHO) declared the Ebola epidemic in West Africa as a Public Health Emergency of International Concern, urging states with intense Ebola Virus Disease (EVD) transmission to ensure that “treatment centres and reliable diagnostic laboratories are situated as closely as possible to areas of transmission” [1, 2]. By that time, in Sierra Leone there were more than 900 confirmed cases and 380 deaths attributed to EVD [3]. In addition to the lack of infection prevention control (IPC) measures in existing healthcare facilities [4, 5], the existence of few and distant Ebola healthcare facilities and a shortage of ambulances [6, 7] contributed to limited access on quality care.

The first suspected case of EVD was identified in Tonkolili district, Sierra Leone, in late July 2014. The district general hospital in Magburaka town (capital of Tonkolili and largest town in the district) functioned as a holding centre for testing of suspected Ebola patients. Prior to their transportation to an Ebola Management Centre (EMC), many patients were admitted to either i) the district holding centres or ii) the district community care centres (CCC—basic health care isolation units where suspected and probable cases of EVD were treated awaiting their test results) [8]. Between July and December 2014, the lack of a local EMC in Tonkolili district obliged all Ebola patients to be transported to EMCs in other districts. The closest functional EMCs to Tonkolili district in that period were the Médecins Sans Frontières (MSF) EMCs in Kailahun and in Bo (269 and 100 km from Magburaka town, respectively). Patients testing positive for EVD in Tonkolili, as well as suspected cases who had not had a confirmatory blood test, were referred to one of these two facilities depending on bed availability. From August 2014, the MSF EMC in Kailahun started receiving an increasing number of ambulances transferring suspected Ebola patients from Tonkolili district. After opening in late September 2014, a similar trend was observed at the Bo MSF EMC from mid November 2014. Interviews conducted with patients who were transferred from Tonkolili district revealed appalling road and travel conditions, long journey times and delays in transfer to Kailahun and Bo EMCs (personal communication GC). Holding centres were described as crowded (some patients reported sharing the same bed with another patient) and had few staff, resulting in long hours before dead bodies were removed. Patients reported spending a minimum of one day and a maximum of 14 days in those centres. Referrals depended on the severity of illness. Patients expressed concerns about the long distance, bad roads, crowded ambulances (6–11 patients), lack of food and water during travel, and lack of bodily fluid disposal (for vomit, diarrhoea). Deaths during transport were also reported (personal communication GC).

The increasing number of patients from Tonkolili in MSFs distant EMCs and the lack of other organisations ready to open an EMC in Tonkolili, led MSF to respond in the district. Thus, inDecember 2014, a new MSF EMC was opened 2 km from the centre of Magburaka town and became the primary referral health facility for Ebola suspected cases in the area. At that time, Tonkolili was one of the six districts with the highest cumulative EVD incidence rates [9].

Early isolation of EVD suspect patients is considered a central pillar for successful outbreak control [10–12], but there is little documentation relating EMC proximity with case fatality and timeliness of admission.

In this study, we aimed to determine whether accessibility of EMC facilities was associated with timeliness of admission and case fatality.

## Methods

We carried out a retrospective cohort study among residents of Tonkolili district who were confirmed EVD cases referred to or admitted in the MSF EMCs in Kailahun, Bo and Magburaka towns of Sierra Leone.

### Definitions

Cases were residents of Tonkolili district who presented to the MSF EMCs between 12 September 2014 and 23 February 2015 and were confirmed as having EVD by positive Ebola polymerase chain reaction (PCR) test of blood or saliva. This included patients who were dead on arrival (DOA) at these facilities and tested positive posthumously. Quantitative reverse transcription PCR results of cycle threshold (Ct) are a measure inversely related to Ebola viral load. Previous analyses indicated high fatality in patients with Ct values ranging from 20.5 to 21.4 [13]. Thus, we considered the viral load high when Ct value was ≤22 [14–16].

Time to admission was defined as the number of days between self-reported symptom onset and admission to the EMC. We defined wet symptoms as vomiting and/or diarrhoea, and dry symptoms as all other symptoms (e.g. headache, fatigue etc.). “Distant” EMCs were those located in Kailahun and Bo, while “district” EMC was the EMC in Magburaka. Transport time was the estimated hours travelling required to reach the EMC from the village of residence of the patient and was based on time records established by the MSF team based in Magburaka.

### Laboratory investigation

Specimens from Bo EMC were sent for EVD confirmation to the onsite laboratory provided by the US Centers for Disease Control and Prevention (CDC), while those from Kailahun and Magburaka EMCs were sent to onsite laboratories provided by the Public Health Agency of Canada (PHAC). CDC and PHAC laboratories used quantitative PCR assays which differed on targeting genes: CDC assays targeted Ebola virus nucleoprotein (NP) and viral protein (VP) 40 [17] while PHAC assays targeted Ebola virus polymerase (L) and NP. [18]. In both laboratories, the specimens were considered positive if the Ct values were ≤40 [15].

### Data collection and analysis

Doctors and nurses triaged patients arriving at the MSF EMCs using the standard EVD case definition, and admitted to the EMC for confirmation those meeting the suspected case criteria. Triage information was collected using the CDC standardised case investigation form in use routinely in Sierra Leone during the outbreak. This included information on demographics, village and district of origin, onset of symptoms, clinical information (presence and type of symptoms), and risk factors (contact with sick people, attending funerals, previous hospital or holding centre admission, travel history). Admission data were entered by an epidemiologist in a specifically designed linelist in Microsoft Excel. For patients previously tested, those laboratory results were also recorded. Additional information was recorded during admission including laboratory test results and final patient outcome. DOA patients were not admitted to the EMC, so their details were recorded in a separate list including laboratory test results, and as much information as was available on age, sex and district of origin; most other variables were unknown for those patients. Cases alive and DOA coming from Tonkolili District were identified from the line-lists and the DOA list, respectively, of each MSF-operated EMC.

We described the cohort of all the cases, including the DOA cases. We measured EMC specific case-fatality as the number of deaths in EVD positive cases, out of the total number of confirmed EVD cases admitted to the EMCs. To examine associations between fatality, viral load, time to admission and the variables of age, gender, chiefdom of residence, EVD symptoms, transport time, distance in kilometres, and EMC of admission, we considered statistical significance at *P* value ≤ 0.05 level and calculated crude and adjusted Risk Ratios (aRRs) for fatality and delayed admission (>3 days after symptom onset) using binomial regression, and 95% confidence intervals (95%CI). We constructed initial regression models including all variables for which (i) the p-value (for the RR) was less than 0.20, or (ii) the RR was more than 1.1 or less than 0.9 in the univariate analysis (corresponding to a 10% change in risk). To simplify the models, we removed variables one at a time depending on the significance testing (p<0.05) by the likelihood ratio (LR) test. We used linear regression to examine associations with normally distributed continuous variables. To identify factors associated with time to admission (not normally distributed), we used generalized linear models assuming a gamma distribution and a logarithmic link. We analysed data using Stata (version 12, Stata Corporation, Texas, USA).

### Ethical issues

The study used data collected for clinical purposes during the emergency outbreak response, and anonymised prior to analysis. The study was conducted as part of the post-outbreak evaluation and met the criteria of the MSF Ethics Review Board for exemption from ethics review for retrospective analyses of routinely collected programmatic data [19].

## Results

Overall, 372 residents of Tonkolili presented to the three MSF EMCs: 210 (56%) to Kailahun, 118 (32%) to Magburaka and 44 (12%) to Bo. Of those, 360 were admitted and 12 were dead on arrival (DOA). Of those admitted, 187 (52%) were female; the median age was 29 years (IQR 18, 39); 40 (11%) were children up to 5 years of age, of which 33 (83%) were referred to distant EMCs. Admitted patients came from all 11 chiefdoms of Tonkolili, with 47% (n = 160) coming from Kholifa Rowala, the second most populated chiefdom in the district.

Of all admitted patients, 249 (69%) were confirmed by PCR as EVD cases; 139 (56%) were female; the median age was 28 years (IQR 17–38). The descriptive and regression analyses focused on the 249 confirmed admitted EVD cases. Of all confirmed EVD cases, 166 (67%) were admitted to Kailahun EMC from 12 September to 30 November 2014, 40 (16%) were admitted to Bo EMC from 4 November to 21 December 2014, and 43 (17%) to Magburaka EMC from 15 December 2014 to 21 March 2015 (Table 1). Thus, of all patients who presented to EMCs, including those DOA, 83% (174/210) were confirmed EVD cases in Kailahun, 95% (42/44) in Bo, and 36% (43/118) in Magburaka.

**Table 1:** Characteristics of Ebola Virus Disease (EVD) confirmed patients from Tonkolili, admitted in MSF Ebola Management Centres (EMCs), Sierra Leone.

Characteristic	Category	Total n (%)†† N = 249	Bo (Distant EMC) n (%)†† N = 40	Kailahun (Distant EMC) n (%)†† N = 166	Magburaka (District EMC) n (%)†† N = 43	Distant EMCs n (%)†† N = 206	p-value*
**Age group (years†)**	≤5	25 (10)	5 (13)	18 (11)	2 (5)	23 (11)	0.46
	6–18	51 (21)	5 (13)	35 (21)	11 (26)	40 (20)	
	19–45	142 (57)	23 (58)	93 (56)	26 (60)	116 (57)	
	46–79	30 (12)	7 (18)	19 (12)	4 (9)	26 (13)	
**Sex**	Female	139 (56)	19 (48)	99 (60)	21 (49)	118 (57)	0.31
**Diarrhoea**	Yes	125 (63)	12 (52)	91 (69)	22 (53)	103 (66)	0.09
**Vomiting**	Yes	119 (60)	11 (48)	88 (66)	20 (49)	99 (63)	0.09
**Wet symptoms**	Yes	159 (80)	17 (71)	113 (84)	29 (69)	130 (82)	0.06
**Transport time**	≤2hours	42 (20)	3 (7)	0 (0)	39 (93)	3 (7)	<0.001
**Time to admission**	>3 days	163 (74)	32 (84)	115 (80)	16 (41)	147 (81)	<0.001
**PCR Cycle threshold**	**≤22**	58 (24)	10 (30)	37 (22)	11 (26)	47 (24)	0.08

* Compares district vs distant EMCs.

† One case in Kailahun EMC did not have an age recorded.

†† column percentages; denominators change due to missing values.

Malaise (90% n = 178), fever (83% n = 134), diarrhoea (63% n = 125), headache (73% n = 140) and vomiting (60% n = 119), were the most frequent symptoms of cases reported at admission. Of all confirmed cases, 80% presented with wet symptoms, with or without dry symptoms; 69% in Magburaka EMC, 71% in Bo, and 84% in Kailahun.

### Dead on arrival

Of 12 patients who were dead on arrival, 10 (83%) were later confirmed as cases: 8 in Kailahun and 2 in Bo. Two DOA patients had no record of being tested: one in Kailahun and one in Magburaka. Of all DOA cases, 50% were male.

### Time to admission

The estimated median transport time from cases’ residences to the EMCs was 4 hours (IQR 2,5 hours), significantly less for the district EMC (1 hour) compared to the distant EMCs (5 hours) (p<0.001) (Table 2). Of all the cases with transport time ≤ 2 hours (n = 42), 93% had been admitted in the district EMC (Table 1).

**Table 2:** Median age, time to admission and transport time of Ebola Virus Disease (EVD) cases from Tonkolili, Sierra Leone, by MSF Ebola Management Centres (EMCs).

Characteristic	Total Median (IQR**)	Bo Median (IQR**)	Kailahun Median (IQR**)	Magburaka Median (IQR**)	Distant EMCs Median (IQR**)	p-value*
**Age (years)**	28 (17,38)	26 (19,41)	29 (17,37)	30 (15,40)	27 (17,37)	0.56
**Time to admission (days)**	6 (3,8)	7 (4,8)	6 (4,8)	3 (2,7)	6 (4,8)	<0.001
**Transport time (hours)**	4 (2,5)	3 (3,3)	5 (4,5)	1 (1,2)	5 (3,5)	<0.001
**PCR Cycle threshold**	26 (20,32)	26 (21,29)	28 (22,32)	24 (19,32)	27 (16,38)	0.03

*compares district EMC (Magburaka) vs distant EMCs (Kailahun and Bo).

**IQR = interquartile range.

The median time from symptom onset to admission was 6 days (IQR 4,8) in distant EMCs compared with 3 days (IQR 2,7) in the district EMC (p<0.001) (Table 2). The mean age of cases with time to admission ≤ 3 days (25.1 ± 15.5; n = 57) was lower, but not significantly (p = 0.08), compared with that of cases with longer (>3 days) time to admission (29.1 ±14.9, n = 162).

Cases with time to admission >3 days after symptom onset were 2.0 (95% CI 1.4–2.9) times more likely to be admitted to the distant EMCs (81%), compared with the district EMC (41%) (Table 1, Table 3). Findings were similar when a 2-day cut-off value for time to admission was used or when time to admission was treated as a continuous outcome variable (data not shown).

**Table 3:** Time to admission (>3 days) to Ebola Virus Disease (EMC) by selected characteristics, Tonkolili, Sierra Leone, 2014–2015, (final binomial regression model).

Characteristics	Category	Patients with time to admission (>3 days)			Adjusted Risk Ratio (aRR)	95% CI
		n	Total	(%)		
**Age (years)**	≤5	11	17	65	Ref	
	6–18	30	47	64	1.1	0.7–1.7
	19–79	121	155	78	1.3	0.9–2.0
**EMC**	Magburaka*	16	39	41	Ref	
	Kailahun	115	144	80	2.0	1.4–2.9
	Bo	32	38	84	2.1	1.4–3.1
	Distant EMCs*	147	181	81	2.0	1.4–2.9
**PCR Cycle threshold**	≤22	31	50	62	0.8	0.6–1.0
	>22	125	162	77	Ref	

*Distant EMCs = Kailahun and Bo; District EMC = Magburaka.

### Viral load and time to admission

Among all cases, the median Ct value was 26 (range 14–38) (Table 2). The median Ct value of cases presenting to the district EMC (24; range 14–30) was significantly (p = 0.03) lower compared to those presenting to the distant EMCs (27; range 16–38). The Ct value increased by 0.29 (95% CI 0.12–0.45; p = 0.018) per day increase in the time to admission, after adjusting for age and bleeding at admission (final linear regression model). Similarly, the mean Ct value (25.0 ± 5.3; n = 56) of cases with time to admission ≤3 days after symptom onset was significantly lower compared to that of cases with longer time to admission (27.3 ± 5.5; n = 156; p = 0.0085). Cases with time to admission >3 days were less likely to have a high viral load (Ct ≤22) (aRR 0.8; 95% 0.6–1.0 compared to Ct>22) (Table 3).

### Factors associated with fatality

Of the admitted cases, 110 (44%) died in the three EMCs: 70 (42%) in Kailahun, 20 (47%) in Magburaka and 20 (50%) in Bo (Table 4). Case fatality was highest in those ≤ 5 years of age (68%). On unadjusted analysis, case fatality was 2.4 (95%CI 1.7–3.4) and 1.5 (95%CI 1.1–2.2) times higher among those with time to admission ≤ 1 day or 1–3 days, respectively, compared with those with longer (≥4 days) time to admission (Table 4).

**Table 4:** Case fatality, (N = 110) according to selected characteristics, Tonkolili, Sierra Leone, 2014–2015.

Characteristics	Category	Deaths	Cases	Case-fatality (%)	Risk Ratio (RR)	95% CI	Adjusted RR (aRR)	95% CI
**Age (years)**	**≤5**	17	25	68	1.7	1.2–2.4	-	-
	**6–18**	20	51	39	1.0	0.7–1.5	-	-
	**19–45**	56	142	39	Ref	–	-	-
	**46–79**	16	30	53	1.4	0.9–2.0	-	-
**Sex**	**Male**	54	106	51	Ref	-	-	-
	**Female**	56	138	39	1.3	1.0–1.7	-	-
**EMC**	**Magburaka**	20	43	47	Ref	-	-	-
	**Bo**	20	40	50	1.1	0.7–1.7	-	-
	**Kailahun**	70	166	42	0.9	0.6–1.3	-	-
**Time to admission (days)**	**4–21**	56	165	34	Ref	-		-
	**1–3**	24	46	52	1.5	1.1–2.2	-	-
	**≤ 1**	9	11	82	2.4	1.7–3.4	-	-
**Vomiting**	**No**	26	79	33	Ref		-	-
	**Yes**	50	119	42	1.3	0.9–1.8	1.4	1.0–2.0
**PCR Cycle threshold**	**>22**	63	185	34	Ref	-	-	-
	**≤22**	47	58	81	2.4	1.9–3.1	2.6	1.8–3.6

Bo and Magburaka EMCs admitted higher proportion of cases with Ct value ≤22 (30% and 26%, respectively), compared with Kailahun (22%) (Table 1). High viral load (Ct≤22) at admission (aRR = 2.6, 95%CI 1.8–3.6) and presenting with vomiting (aRR = 1.4, 95%CI 1.0–2.0) were the two factors significantly associated with a fatal outcome in the final binomial regression model (Table 4).

## Discussion

The study provides evidence that the opening of the district EMC in Tonkolili, Sierra Leone, was associated with reduced DOA and admission delays, with time to admission significantly lower for cases admitted in the district EMC compared with distant EMCs. Other studies also refer to the potential spread of the virus from patients before they were admitted to an EMC [20, 21, 22]. Patients originating from distant districts may have contributed to Ebola virus transmission locally due to the longer time spent home before seeking care and the need to traverse communities to reach a distant EMC [7, 16].

Case fatality did not differ significantly between district and distant EMCs. However, those admitted to the district EMC had higher viral load (lower Ct) at the time of admission compared with those admitted to the distant EMCs. High viral load has been used to indicate severe disease [23]. In this study, high viral load was identified as a significant risk factor for death. Other studies found a sharp increase in case fatality from 21% in patients with low viral load to 81% in those with high viral load [24, 25]. Our study did not include undetected cases and deaths in the community or holding centres who did not present to an MSF EMC. It is therefore possible that patients in the community with severe EVD symptoms did not manage to reach a distant EMC as they may have died quickly, introducing a survival bias [2, 7, 16].

Our study indicated that viral load decreased with increasing time to admission. This is consistent with another study that observed a rapid decline of viremia in those still alive after the first week since onset of symptoms [23]. In our cohort, cases admitted to the distant EMCs had longer time to admission. Patients reported spending several days in holding or CCC before being referred. Thus, viremia may have declined by the time of admission to the distant EMCs.

The high fatality in those reaching the EMCs within the first days of symptom onset could also be explained by the high-level viremia among patients with early admission. Early admissions were more likely in the district EMC. This suggests that, although early admission might play a critical role to interrupt community transmission, it might not contribute to substantially reduced fatality in patients presenting with high levels of viremia. However, other authors documented the double benefit of increasing survival and interruption of transmission once rapid response occurred at community level, ensuring timely detection of cases [26].

In our study, the proportion of positive patients admitted differed across the three EMCs, possibly due to different admission criteria of those EMCs. Bo EMC accepted almost entirely patients already confirmed positive. Kailahun EMC opened early in the outbreak and accepted any suspected case. However, patients reported that only those with the most severe symptoms were referred to Kailahun from Tonkolili. This is reflected in the high proportion of confirmed cases. Magburaka EMC accepted any suspected case, in a period when more EMCs were available in other districts and community awareness may have been better, thus the triage was more sensitive compared to the distant EMCs.

Increased transport and bed capacity in the district EMC, allowing safe isolation of cases, made dedicated care accessible to the district population; this may have reduced infectious time in the community for cases residing in Tonkolili.

The vast majority of the cases with transport time ≤ 2 hours had been admitted in the district EMC. The EMC in Magburaka, located closer to the district population, admitted patients soon after their onset of symptoms, suggesting that patients sought care early when care was available within a short travel time. Patients reported that travel to distant EMCs involved lack of food, water, and the ability to safely dispose of human fluid (personal communication GC). This could have contributed to 12 patients being dead on arrival following long arduous journeys, with this outcome only occurring for confirmed cases travelling to distant EMCs.

Long transfers had other negative consequences. Patients with suspected EVD were transported for long distances in ambulances, sometimes up to 11 patients per ambulance. Some of those transferred were found to be EVD negative after testing on arrival at the EMC [12]. The exposure such patients may have had from sharing an ambulance with EVD positive, usually symptomatic patients, may have resulted in Ebola transmission during transfers [16]. Many patients also reported not having been informed of their final destination or about their prospects of receiving adequate care (personal communication GC). In addition, families were only informed many months later of their relatives’ final outcomes. Similar experiences were reported in Liberia, where families referred to EMCs as “black holes” where sick family members disappeared [27].

The EMC in Tonkolili district was constructed by MSF within 18 days from the time the decision was made to open an EMC in the district [28]. However, due to delays in making the decision to open an EMC in this site, less than one fifth of Tonkolili’s known Ebola case burden was admitted to the district EMC. The impact of the district EMC on the control of the epidemic in the region might have been greater if it had been available in October 2014, when cases from the district peaked, or in July 2014 when the first cases appeared in Tonkolili district. By serving as the primary referral point to receive suspected EVD cases, the Tonkolili EMC, an EMC with standard Ebola IPC procedures, may have reduced ambulance and nosocomial transmission by reducing journey time, and removed the need for referrals to distant EMCs. Additionally, its proximity facilitated communication with the families of the admitted or DOA patients, which was more difficult to achieve in distant EMCs.

In our study, we observed a significant association between increased fatality and both high viral load and vomiting at admission, which is consistent with other studies [7, 13–16]. In addition, many patients referred to the distant EMCs had first been admitted to a holding centre or a CCC, often for a few days [8].

### Limitations

The case numbers and case fatality rate represent only those patients who arrived at one of the three MSF EMCs, thus excluding undetected cases and deaths in the community or holding centres.

In the analysis, we have not adjusted for calendar time; patients arrived in each one of the EMCs in different calendar times, as the district EMC opened months after the distant EMCs. So, results might have been influenced by other, unaccounted factors.

Some dates of symptom onset may not have been accurately recorded, due to the severe condition of patients at admission, which in some cases allowed very little clinical history to be obtained. However, if patient condition stabilised during admission, clinical staff re-interviewed those patients to elicit more accurate information.

Being in a complex emergency context, we could only record minimal information on the DOA patients. Those patients arrived by ambulance unaccompanied without any relative present. Therefore, we could not collect information on time to admission and clinical characteristics that would help us examine further the association between DOA and proximity to EMC.

As this study utilised only minimal data routinely collected as part of EMC management, we could not measure several factors that may be associated with fatality, such as level of care and comorbidities. In addition, we could not account for other changes in the response to the Ebola outbreak, including community attitudes and behaviours, which may have influenced the speed of identification of suspected Ebola cases.

Other factors apart from EMC proximity, may have contributed to reduced time to admission. Studies have documented that improvements in surveillance, more effective contact tracing and availability of ambulances might play a role in reducing delays in accessing care [29]. However, we did not have information on these factors for all EMCs in which patients were referred. Therefore, we could not adjust for these factors in analysis. But program reports from agencies involved in the response suggest that the best serviced in terms of these factors were also the districts with closer EMCs.

### Conclusions and recommendations

The availability of a central district EMC was associated with reduced DOA and delays of admission to care of EVD patients during the Ebola outbreak in Tonkolili district, Sierra Leone in 2014–15. Potential benefits of this are likely to have been reduced community and ambulance and nosocomial transmission, along with more standardised healthcare provision. In any future Ebola outbreak with established transmission in the community, health authorities should consider strategic location of EMCs as an important component of a successful response strategy, taking into consideration availability of adequate health staffing, logistics, essential medicines and medical supplies.

## Abbreviations

aRRs: adjusted Risk Ratios
CCC: Community Care Center
CDC: Centers for Disease Control and Prevention
CI: Confidence Interval
Ct: Cycle threshold
DOA: Dead on Arrival
EMC: Ebola Management Center
EVD: Ebola Virus Disease
IQR: Interquartile Range
IPC: Infection Prevention Control
MSF: Mèdecins Sans Frontiéres
PCR: Polymerase Chain Reaction
PHAC: Public Health Agency Canada
